# Modelling of Mechanical Behavior of Biopolymer Alginate Aerogels Using the Bonded-Particle Model

**DOI:** 10.3390/molecules24142543

**Published:** 2019-07-12

**Authors:** Maksym Dosta, Kolja Jarolin, Pavel Gurikov

**Affiliations:** 1 Institute of Solids Process Engineering and Particle Technology, Hamburg University of Technology, 21073 Hamburg, Germany; 2 Institute of Thermal Separation Processes, Hamburg University of Technology, 21073 Hamburg, Germany

**Keywords:** mechanical behavior, plastic deformation, aerogels, discrete element method, bonded-particle model

## Abstract

A novel mesoscale modelling approach for the investigation of mechanical properties of alginate aerogels is proposed. This method is based on the discrete element method and bonded-particle model. The nanostructure of aerogel is not directly considered, instead the highly porous structure of aerogels is represented on the mesoscale as a set of solid particles connected by solid bonds. To describe the rheological material behavior, a new elastic-plastic functional model for the solids bonds has been developed. This model has been derived based on the self-similarity principle for the material behavior on the macro and mesoscales. To analyze the effectiveness of the proposed method, the behavior of alginate aerogels with different crosslinking degrees (calcium content) was analyzed. The comparison between experimental and numerical results has shown that the proposed approach can be effectively used to predict the mechanical behavior of aerogels on the macroscale.

## 1. Introduction

In an effort to tackle pollution in seas, fields and waterways, regulatory authorities support a wide-ranging ban on single-use plastics. The trend towards minimizing our dependency on synthetic crude-oil derived plastics is also echoed by the chemical industry, both in Europe and worldwide. One common solution currently in research and development is the use of natural and renewable sources of biopolymers. Examples are marine biopolymers (alginate, agar, chitin and chitosan) and plant-derived biopolymers such as cellulose, starch, lignin pectin and proteins. The biopolymers are generally recognized as the most sustainable starting molecules for beeing converted into functional material alternatives to plastics.

Although some difficulties in their use, such as variable molecular weight, impurities and lability against microorganisms, can successfully be overcome [1], the area of high-performance materials is traditionally thought to be the least prone to changes. In particular, lightweight thermal and sound insulation (for clothing, buildings, aircrafts, packaging) has been dominated essentially by polyurethane- and polystyrene-based synthetic materials.

In 2014–2015 it was reported that cellulose and alginate can be converted into highly porous nanostructured materials—aerogels—with exceptionally low thermal conductivities below that of air (26 mW/m·K), see Rudaz et al. [2], Gurikov et al. [3], Kobayashi et al. [4]. Shortly after these publications, this fact was confirmed for other biopolymers such as pectin and chitosan [5]. These achievements showed that even high-performance lightweight insulating materials could be of purely biopolymer nature. However, biopolymer aerogels often possess poor mechanical performance impeding their further development as functional materials. This problem has been tackled from chemical perspectives [6,7,8] but mechanical performance of aerogels in relation to the structural features is still far from being understood.

Many biopolymer aerogels have qualitatively similar fibrillar structures, making them very different from classical oxide-based aerogels with interconnected nanoparticles. For classical oxide-based aerogels a substantial body of experimental data have been reported and a wide range of modelling methods, including all-atom molecular dynamics, coarse-grained and macroscopic models, have been suggested [9,10,11,12,13,14]. Mechanical tests, primarily axial compression tests, for fibrillar biopolymer aerogels only recently started to appear in the literature [15]. To the best of our knowledge, only one approach towards modelling of mechanical properties of biopolymer aerogels, the so called micromechanical constitutive model, has been proposed by Rege et al. [16,17,18]. This model considers the cellular network of biopolymer aerogels to be made up of continuously and isotropically distributed idealized square shaped microcells. The macroscopic compressive deformation is dictated by the bending of the cell wall fibrils at the microscale. The overall macroscopic predictions of the model have been shown to be in good agreements with the uniaxial compression data for several biopolymers.

Another modelling approach is the bonded-particle method (BPM). It has been developed as an extension of the discrete element method (DEM) for modelling of granular materials [19]. A material modeled by the BPM is represented as a set of discrete primary particles connected with solid or liquid bonds. Each bond connects two primary particles and depending on the relative motion of the connected particles forces and moments emerge in the bond. These forces and moments are finally acting on primary particles and leading to their translational and rotational motion. In each time step additional breakage criteria are analyzed. If one of the criteria is fulfilled the bond is destroyed and removed from the simulation domain.

In recent years BPM has been widely applied for modelling of different types of materials [20] ranging from glass agglomerates [21] to concrete [22] or sandstone [23]. It is also applicable for a wide range of scales. By coupling the BPM with a finite-element simulation Wu et al. [24] successfully applied the BPM even to structural-scale boundary value problems like borehole instabilities. Often, experimental data on internal structure and sample shape is used to generate the structural model in BPM. In many cases data obtained from micro computed tomography (μCT) can be effectively employed. For automated approximation of the material structure from μCT data sets, a bonded-particle extraction technique was developed [25]. From a rheological point of view in most cases purely elastic materials are modeled with BPM. In some contributions authors have applied this approach for modelling of plastic behavior. Nguyen et al. [26] has developed a combined damage-plasticity cohesive model for cement bridges in soft rocks, for modelling of asphalt concrete. Kim and Buttlar [27] have proposed bilinear cohesive model, Tran V.T., Donze F.V., Marin P. [28] have applied a elastic-hardening-damage law to reproduce irreversible compaction for concretes under high confining pressure. For the simulation of failure behavior of quasi-brittle materials Ma et al. [29] proposed a displacement-softening contact model.

However, until now the BPM was not applied for the modelling of highly porous plastically deformable materials such as biopolymer aerogels. In this contribution we extend the BPM with a new functional model for solid bonds and apply it for the modelling of the mechanical behavior of alginate aerogels.

## 2. Results

### 2.1. Experimental Characterisation

#### 2.1.1. Mechanical Behavior

**Figure 1:**
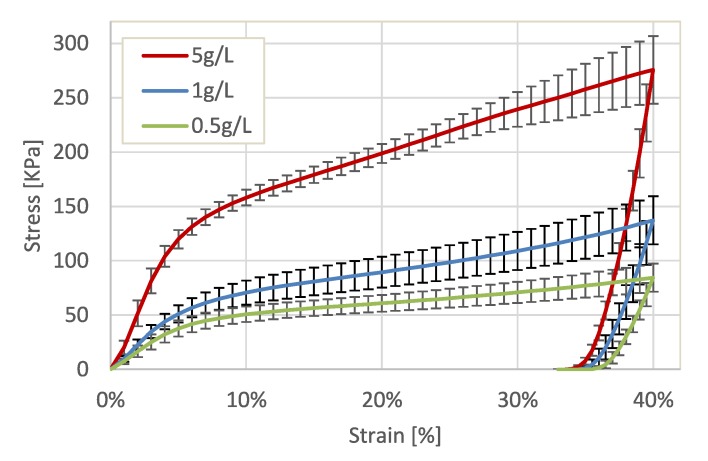
Averaged stress-strain characteristics for alginate aerogel samples synthesised with different CaCl${}_{2}$ concentrations.

To characterize the mechanical behavior of the well-shaped cylindrical biopolymer aerogels samples (see Section 4.1), quasi-static uni-axial compression tests were used. The tests were performed with the Texture Analyzer device from Stable Micro Systems Ltd., Surrey, United Kingdom (force resolution: 0.1 g, displacement resolution: 0.001 mm). The cylindrical samples were oriented on a steel platform in vertical direction and were loaded with a cylindrical steel punch. The punch was moved downwards with a constant velocity of 0.1 mm/s. A strain equal to 0.4 was specified as a stop criterion. For each of the three types of aerogels crosslinked with different CaCl${}_{2}$ concentrations, several samples have been loaded to obtain average characteristics. The number of analyzed samples was equal to 19, 20 and 16 for the concentrations 0.5 g/L, 1 g/L and 5 g/L, respectively. The corresponding averaged stress-strain curves along with standard deviations for all samples are shown in Figure 1. To convert forces into compressive stresses, the measured cross-cut radius was used.

During compression of the aerogels two main regions can be generally distinguished: elastic deformation and plastic yielding. The initial region of elastic deformation is followed by the plastic yielding which is started for strains above 4%.

From analysis of the unloading phase it can be observed that samples with high CaCl${}_{2}$ concentrations have slightly larger elastic relaxation compared to the samples with lower CaCl${}_{2}$ content. In case of 5 g/L, the recovered elastic deformation equals to almost 6% of initial height. Contrary to this, for samples with a concentration of 0.5 g/L the elastic recovery is about 3.5%.

**Figure 2:**
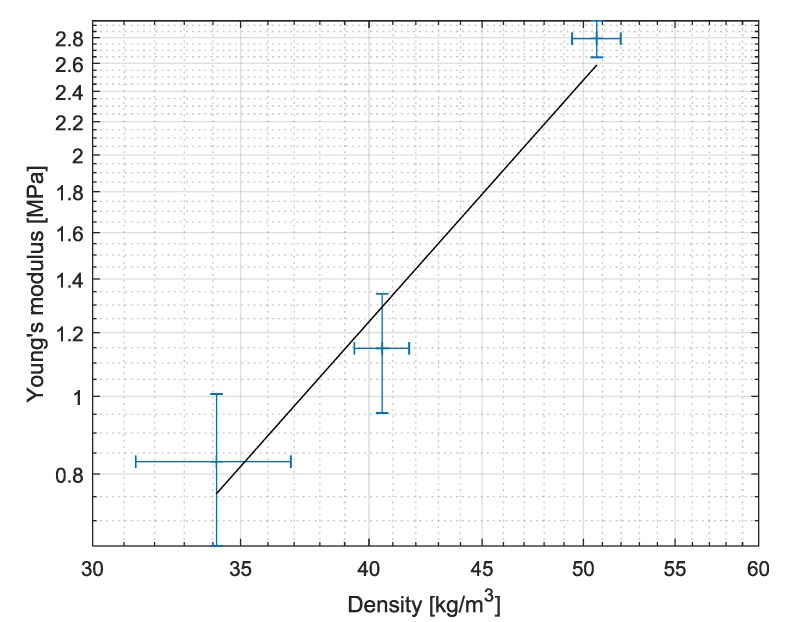
Dependency of Young’s modulus on material density.

Considering the influence of the crosslinking degree on the mechanical behavior, Figure 1 shows that, more stronger crosslinked aerogels have a higher stiffness, as expected. To characterize the materials, the Young’s modulus has been extracted from the experimental results. For this purpose, the slope of the force-displacement characteristics in the region of elastic deformation, namely between 1% and 4% strains, was analyzed. The dependency between strain and stress in this region is almost linear within experimental uncertainties. In Figure 2 the calculated Young’s modulus (*E*) is plotted versus the material density ρ. This dependency can be effectively described with a power-law relationship.

(1) $$\ln\left(E\right)=-11.25+3.11\ln\left(\rho \right)\;.$$

To analyze the material behavior under different type of mechanical stresses, additionally to the uni-axial compression test, three-point bending tests have been carried out. The Texture Analyzer three-point bend rig HDP/M3PB was been used for the measurements. Detailed description of the performed experiments and obtained results is given in Section 2.2.2.

#### 2.1.2. Deformation and Densification

**Figure 3:**
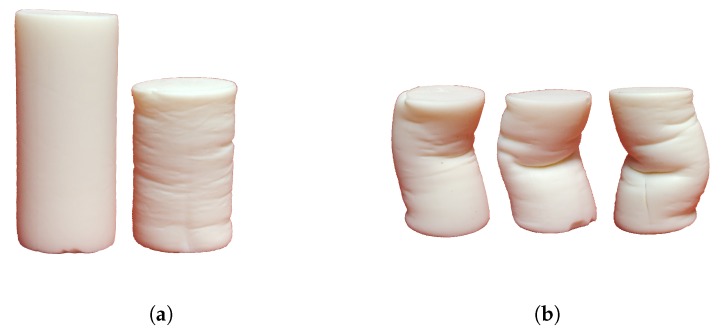
Deformation behavior of aerogel samples: (**a**) Initial and compressed sample with local buckling (wrinkling) effects, (**b**) Global buckling effects.

Significant buckling effects were observed during material compression. To characterize material deformation, samples before and after compression have been compared visually. In Figure 3 initial sample and typical deformation behavior are illustrated. In compressed samples global and local buckling effects have been observed. However, in the scope of this work, no detailed investigations of deformational behavior of aerogel samples have been done.

**Figure 4:**
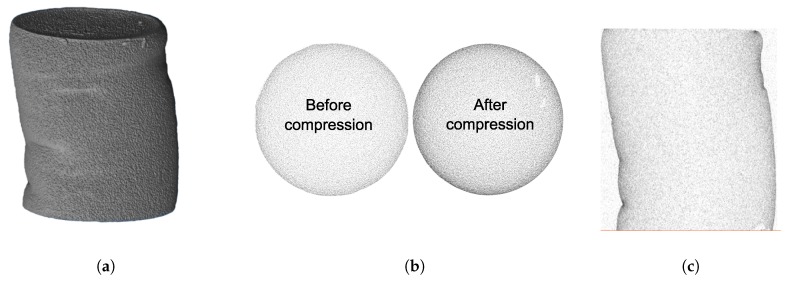
Reconstructions obtained from μCT analysis for alginate aerogel crosslinked with a CaCl${}_{2}$ concentration 5 g/L. (**a**) 3D volume reconstruction, (**b**) horizontal cross-cut and (**c**) vertical-cross cut of material.

To analyze the possible formation of cracks or formation of zones with large density gradients the compressed samples were analyzed using micro computer tomography (μCT). The μCT 35 of SCANCO Medical AG was used to characterize the material. The resolution of this apparatus does not allow to distinguish single micro- and mesopores, however, it allows to observe zones with different densities. In Figure 4 the tomographic reconstruction of sample crosslinked with 5 g/L CaCl${}_{2}$ is illustrated. On the left-hand side a three-dimensional reconstruction of a compressed cylinder after unloading is shown (Figure 4a). In Figure 4b a horizontal cut for a sample before and after loading is illustrated and in Figure 4c a vertical cut for a compressed sample is shown.

Analysis of the μCT images shows that for all aerogels no cracks or large destructions have appeared within the bulk phase. The material demonstrates ductile behavior on the macroscale and large plastic deformations. From comparison of the cross-cuts before and after compression a significant densification can be observed. However, the relative radial change in density in Figure 4b appears to be nearly the same and only the absolute value has changed. Therefore, the images suggest that the densification occurs mainly in vertical direction. Although the original samples also show a certain level of peripheral densification (often referred as to “skin” and most likely originating from partially collapsed pores due to adsorption of air moisture), the skin become much more visible upon compression which can be seen both in the horizontal and vertical cross-cuts (Figure 4b,c).

### 2.2. Simulation Results

#### 2.2.1. Uni-Axial Compression

To test the capabilities of the developed simulation model, a uni-axial compression test was simulated for each of the three different calcium chloride concentrations. To reproduce samples with different concentrations only the Young’s modulus of the bonds and particles were modified according to the derived power-law relationship for the aerogel density (Equation (1)). All other model parameters, for example, the compressive yield strain or the plastic failure, were kept constant. For those parameters the values calibrated for the case of a concentration of 5 g/L were used. In order to avoid numerical errors caused by the stochastic structure generation process, five samples for each concentration were generated.

**Figure 5:**
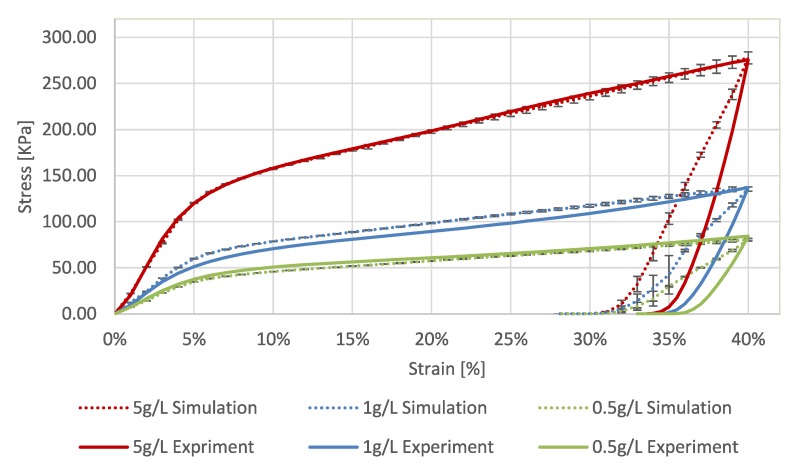
Comparison between experimental and simulation results.

The resulting stress-strain curves obtained from the DEM simulations are shown in Figure 5 along with experimental data. The main features described in previous section, that is, the elastic range and the plastic yielding are predicted accurately. However, in contrast to the experiments, the unloading results in the same recoverey strain for all concentrations.

**Figure 6:**
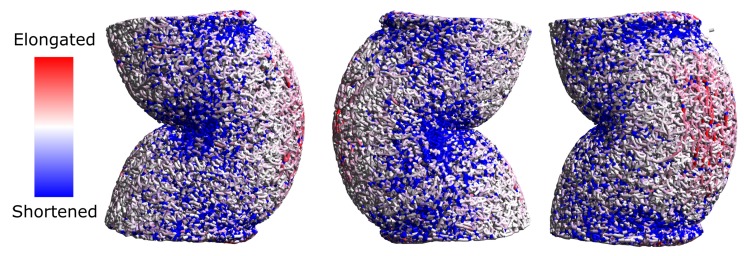
Residual deformation of aerogel samples.

Furthermore, the simulation results show a deformation of the body similar to the experimentally observed buckling. In Figure 6 three typical residual deformations occurring after uni-axial compression are shown. The deformation pattern is very similar to the one experimentally observed (Figure 6). The bonds are colored by the residual strain. In the convex regions prevailed elongated bonds can be observed. Since the functional bond model does not contain tensile plastic deformation, presence of elongated bonds indicates that there is residual stress existing in material. In contrast to the experiments, global buckling effect occured in all simulations. To achieve also a pure compressed state with local wrinkling effects (Figure 3a) in the simulations, a more detailed discretization of the structure is needed.

#### 2.2.2. Three-Point Bending

**Figure 7:**
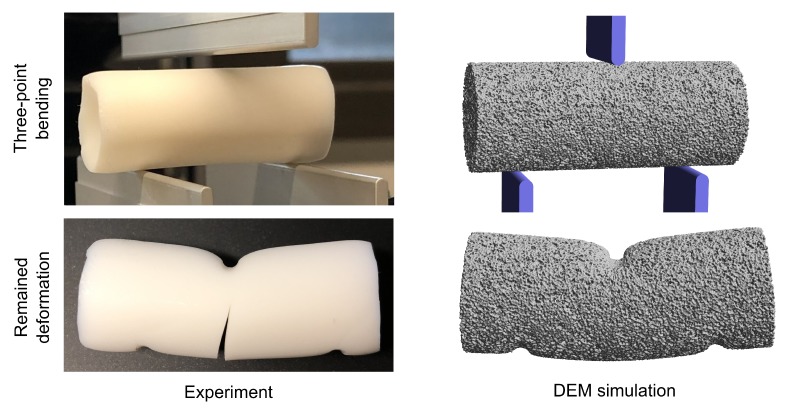
Three-point bending set-up and remained plastic deformation.

To analyze applicability of the model for different type of mechanical stresses, the material behavior during three-point bending tests has been experimentally and numerically analyzed. This has been done for aerogels with a high calcium chloride concentration of 5 g/L. In the upper part of Figure 7 the bending set-up used for experimental and numerical investigations is shown. The distance between the lower punches is equal to 26 mm and their thickness is 2 mm. The diameter of the experimentally analyzed aerogel samples is equal to 15.87 ± 0.08 mm. For the numerical investigations samples with diameter of 15.87 mm were generated.

In the lower part of Figure 7 the typical remained plastic deformations for aerogels are shown. It can be observed that on the one hand material stressing leads to the plastic bending of the whole sample and the formation of a curvature spread over the entire cylinder. On the other hand, in the zones where punches have contact to the sample, large local deformations occur. In these zones large compressive stresses lead to formation of concavities. Both effects are also visible in the numerical results.

Significant deviation between the results is related to the material breakage and initiation and propagation of cracks. Despite the fact, that the model is generally applicable for simulation of material breakage, in the scope of this work no breakage was modelled and the tensile strength was not adjusted. Thus, only in the experimental results crack formation can be observed. In a part of the samples, cracks propagated completely through the material and the sample was destroyed in two or more parts.

**Figure 8:**
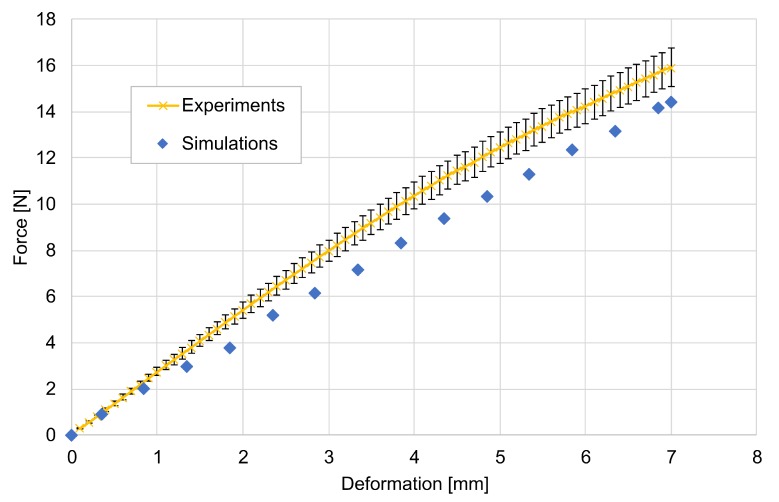
Force-displacement characteristics for three-point bending tests (calcium chloride concentration 5 g/L).

In Figure 8, force-displacement characteristics obtained from the experimental and numerical investigations are illustrated. Since the model parameter for modelling of material failure was not adjusted, that is, the tensile strength of bonds, the experimental and numerical results were compared only up to the breakage point. The elasto-plastic material behavior of aerogels results in the non-linear dependency between the displacement of the upper punch and the measured force. From the comparison in Figure 8, it can be observed, that despite the fact that the proposed model underpredicts the force, it is still able to qualitatively predict the material behavior. Moreover, quantitative comparison shows that the absolute deviations is approximately in the range of 15%.

## 3. Discussion

In the work at hand, a DEM-based model for the investigation of the mechanical behavior of aerogels has been proposed. The model consists of three main subparts: the structural model to represent the internal material structure, the functional model to describe the mechanical behavior of single components and the model parameters which have to be adjusted to the experimental data. The examined biopolymer aerogels consist of pores in the nanometer or sub-micrometer range. However, this was simplified for the modelling. Instead, the porous material structure was represented on the mesoscale as a set of primary particles connected with cylindrical solid bonds with diameters around 100 μm. To describe forces and moments acting in the solid bonds, a new elastic-plastic rheological model was developed. For this purpose, self-similarity of the material behavior on the meso- and macroscales has been assumed. The unknown model parameters were adjusted by using a simplex search optimization method.

To analyze the capabilities of the model, it was applied to cylindrical alginate aerogels samples with varied crosslinking degree controlled by the calcium chloride concentration. The aerogel samples were produced with the following method: cylindrical gels were prepared in dialysis bags immersed in aqueous solution of calcium chloride followed by solvent exchange, polishing and supercritical drying. Uni-axial compression tests were performed to obtain stress-strain curves of the samples. Two different regimes are identifiable: an elastic and a plastic range. The results are in good agreement with general theory of mechanical behavior of foamed structures and reported results for aerogels [29,30]. However, due to the high material porosity, no densification regions were observed for samples with CaCl${}_{2}$ concentrations of 0.5 and 1 g/L. Only for aerogels crosslinked at 5 g/L a slightly pronounced densification region was observed for strains above 35%. Similar results have been obtained by Karadagli et al. [30], where 50% strain was defined as transition stage into the densification region. Also for the dependency of the Young’s modulus on the crosslinking degree (or density) similar correlations as the one found (Equation (1)) have been reported for alginate and other biopolymer aerogels [15].

The experimental results were used as input for the simulations. Moreover, to test applicability of the model for other type of mechanical stressing, three-point bending tests have been performed for samples with high calcium chloride concentration. Analysis of the obtained simulation results has shown that, after adjustment of the model parameters for a specific calcium chloride concentration, the elastic-plastic material behavior can be accurately predicted for all other concentrations with different elasticity moduli. Only the unloading shows discrepancies between the experiments and simulations. They result from the construction of the bond model. Since the yield criteria in the bond model depend only on the strain, the recovered strain does not change with the Young’s modulus. By including a dependency on stresses this could be solved. Nevertheless, the developed model can mimic buckling effects occurring for cylindrical samples and can describe material behavior during three-point bending tests. Overall, we can conclude that the derived elasto-plastic model is capable to reproduce the macro-scale mechanical behavior of the studied aerogel with different crosslinking degrees. Since the fitting was performed only for one crosslinking degree, the parameters of the functional model seem to be material specific. The moderate deviations between experimental and simulation results indicate that the developed DEM-based model can be used for the modelling of aerogels with high efficiency.

## 4. Materials and Methods

### 4.1. Materials

**Figure 9:**
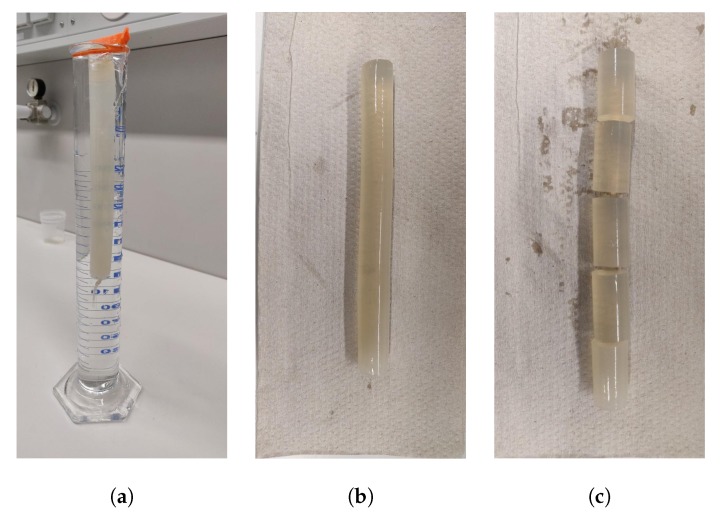
Preparation of cylindrical alginate gel (**a**); alginate gel removed from the dialysis tube (**b**) and cut into cylinders (**c**).

3 wt% alginate solution was prepared using sodium alginate (CAS 9005-38-3, Protanal LF 240 D grade, G/M ratio 0.63:0.37, see Reference [31] for a detailed chracterization). About 40 mL of alginate solution was filled into dialysis tubes of 20 cm length (SERVAPOR${}^{®}$, 16 mm diameter). The tubes were clipped at the top and allowed to stand for about 15 min to ensure that bubbles present in the solution escape to the top. Afterwards the tubes were placed in aqueous solution of calcium chloride. Solutions with concentrations of 0.5, 1.0 and 5 g/L were prepared from crystalline CaCl${}_{2}$·2H${}_{2}$O (CAS 10035-04-8, Carl Roth GmbH, Karlsruhe, Germany). Each bag was placed in a measuring cylinder (250 mL) and then filled with calcium chloride solution (Figure 9a).

In the case of low calcium chloride concentrations (0.5 and 1 g/L) the calcium chloride solution was changed three times every 24 h with a fresh solution of the same concentration to ensure ionic equilibrium.

After complete gelation (Figure 9b), hydrogels were cut into cylinders with a length of ca. 3.5 cm (Figure 1c) to achieve a length-to-diameter ratio of nearly 2:1 after subsequent solvent exchange, supercritical drying and polishing.

The sliced hydrogels were subjected to the solvent exchange with ethanol. It was performed in steps with 30, 60, 90 vol.% ethanol/water mixtures and finally using pure ethanol (containing 1% methyl ethyl ketone; CAS 64-17-5, Carl Roth GmbH, Karlsruhe, Germany). The solvent exchange with pure ethanol was performed thrice to ensure complete removal of water (additionally controlled by density measurements). The gels were immersed in each ethanol grade for at least 4 h. Concaved top and bottom sides were polished by sand paper to obtain flat surfaces.

Finally, the polished alcogels were subjected to dynamic supercritical drying with CO${}_{2}$ at 120 bar, $60{\;}^{\circ }$C for 4 h (approx. 25 g/min flow) to yield aerogels. The aerogels were vacuum dried for 24 h at $50{\;}^{\circ }$C and stored in a desiccator with freshly calcined silica gel beads to minimize contact with moist air.

Before compression tests, the diameter of each cylindrical sample was measured at different heights using a caliper. The averaged diameter was used to estimate the cross-cut surface. The envelope density of the aerogels was estimated from the sample height and the averaged diameter.

### 4.2. Modelling Approach

#### 4.2.1. Bonded-Particle Model

In the BPM, the aerogels are modelled as agglomerates consisting of smaller ideally spherical (primary) particles connected with solid bonds. The bonds are modelled as cylindrical objects with zero mass. Hence, particles can interact directly if they are in contact and they can interact via bonds. Both interactions can occur independently and, therefore, also at the same time between the same particles.

The BPM is a mathematical model which is applied to represent real objects and to mimic their behavior. The model can be subdivided generally into three parts:

- structural model: spatial material distribution, positions and radii of primary particles, connection between primary particles, bonds radii, etc.;
- functional model: functional dependencies to describe forces and moments acting in the bonds and between primary particles;
- model parameters: model parameters to describe plasticity, softening, and so forth.

The structural model is generated in two consequent stages. In the first stage a packing of primary particles was created using a force-biased algorithm. During this process, the particles are randomly placed into a cylindrical volume. The initial number of generated particles was calculated to reach a specified packing density. Afterwards, according to the calculated forces, particles are iteratively shifted to avoid interparticle overlaps. Finally, the radius of the particles is reduced. This results in a homogenous structure, despite the quite low packing density. In the second stage, cylindrical solid bonds are generated between primary particles. If the distance between any two primary particles was less than a specified threshold, the bond is generated. More detailed description of the algorithm can be found in [32,33]. Note that this model is used to represent the material structure on the mesoscale and does not directly consider the nanoporous structure. For the generation of structural models on the nanoscale, other methods like stretching-relaxation method proposed by Kieffer and Angell [34] exist.

#### 4.2.2. Elasto-Plastic Bond Model

The functional model in the BPM consists of three submodels: the particle-particle and the particle-wall interaction model as well as a model for the solid bonds. To calculate contact forces acting between primary particles and between particles and walls the visco-elastic Hertz-Tsuji [35] model for normal force and the Mindlin [36] model for tangential force were applied. The main parameters of the particles is hence the Young’s modulus and the Poisson ratio of the particles as well as the friction coefficient and the rolling friction in case of the tangential forces.

For solid bonds a new elasto-plastic model was developed. At first, we will discuss the general approach and how it was derived. In the second paragraph the details of how it is realised are explained.

For deriving the model, self-similarity of material behavior on the macroscale (cm) and mesoscale (mm) was assumed. Hence, the concept of the elasto-plasic bond model on the mesoscale was derived from the observed macroscopic mechanical behavior. As shown before, in the beginning of the deformation the aerogel reacts linearly elastic. Therefore, we assumed that in this range the internal structure of the aerogel is absorbing the forces. In our model this corresponds to the bonds. Due to the approach for generating the structural model it is ensured that the bonds are dominating at beginning. The experiments showed that at a given point the behaviour transforms into plastic yielding with a lower stiffness. This could correspond to a failure of the internal structure and the rise of forces more similar to interparticle forces. This is modelled by the failure of the bonds and the particle-particle interaction starts to dominate. The measured non-linearity of the slope with increasing strain rates also fits to this assumption. During unloading the aerogel reacts again almost linearly but with a higher stiffness. This is caused by a significant densification of the highly-porous aerogel. As a first approach, this is modelled by an increase of the stiffness.

**Figure 10:**
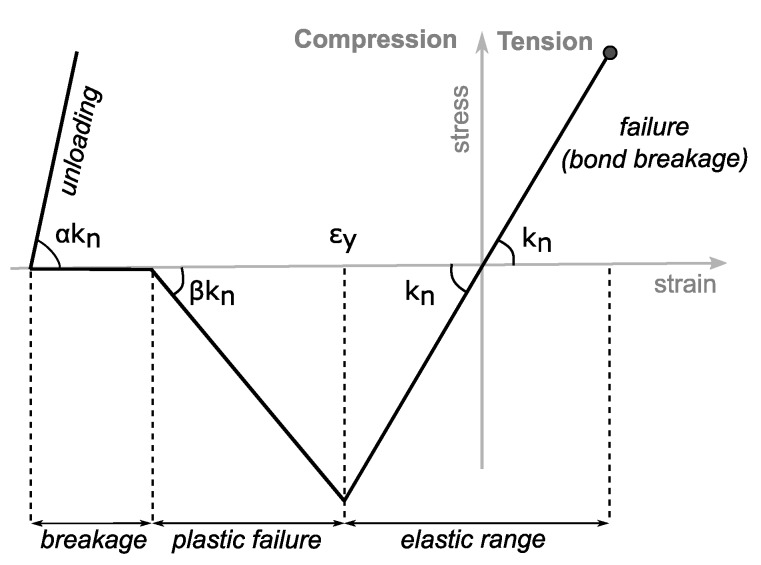
Schematic stress-strain diagram for solid bond loaded in normal direction.

The new bond model is based on the previously proposed linear elastic beam model [20,21]. The forces in tangential direction and the moments were calculated according to this elastic beam model. Only the force in normal direction was calculated by considering the elasto-plastic material behavior. In the calculation, different procedures for tension and compression were used. The schematic stress-strain curves for a single bond under different load conditions are shown in Figure 10.

For both regimes, tension and compression, the model starts as a classical linear elastic beam with the same stiffness

(2) $$k_{n}=\frac{EA}{l}$$

where *A* is the bond cross sectional area and *l* is the initial length of the bond. At a given compressive yield strain $\epsilon_{y}$, the bond fails and the transferred normal force diminishes with $\beta k_{n}$ until it reaches zero. From this point on, no compressive forces are transferred and the bond is treated as quasi-broken bond, however, it is not removed from the simulation scene. Further compression only leads to bond plastic deformation. However, if the particles connected by the bond are in contact, they can still transmit forces according to the functional model for the particle-particle interaction. If the bond starts to be pulled again the tensile stress will act in it. This stress represents interlocking occurring in the structure. The stiffness of a bond in this tensile regime is equal to $\alpha k_{n}$. Thus, unloading of the bond leads to immediate tensile force.

Breakage of the bond due to tensile force is included in the model but was not considered for the work at hand.

#### 4.2.3. Adjustment of Model Parameters

As shown in Figure 10, the used elasto-plastic model has four parameters: the beam stiffness $k_{n}$, the strain rate at which the plastic failure starts $\epsilon_{y}$, the parameters to define the stiffness during the plastic failure β and during unloading α. Since the model is derived heuristically, based on the self-similarity principle, the parameters cannot be easily calculated from theoretical approximations. Instead, a procedure to fit the parameters to the experimental data is proposed. To adjust the parameters, an uni-axial compression test was simulated and obtained strain-stress curves were compared to the experimental results of the compression test. Based on the analysis, a cost function is formulated as sum of two integrals calculated for loading and unloading regimes:

(3) $$F_{\mathrm{err}}=\int_{0}^{\epsilon_{\exp}^{max}}\left|\sigma_{\exp}^{\mathrm{load}}\left(\epsilon \right)-\sigma_{\mathrm{sim}}^{\mathrm{load}}\left(\epsilon \right)\right|d\epsilon +\int_{0}^{\epsilon_{\exp}^{max}}\left|\sigma_{\exp}^{\mathrm{unload}}\left(\epsilon \right)-\sigma_{\mathrm{sim}}^{\mathrm{unload}}\left(\epsilon \right)\right|d\epsilon \;,$$

where $\epsilon_{\exp}^{max}$ is the maximal strain reached in experiments and $\sigma_{\mathrm{sim}},\sigma_{\exp}$ are the stresses obtained from simulation and experiments, respectively. In the first step, the beam stiffness of the bonds $k_{n}$ was adjusted to achieve the measured stiffnesses. In the second step, the model parameters $\epsilon_{y}$, β and α were estimated to fit the curvature of the stress-strain curve. For this purpose, Matlabs (R2018a) fminsearch, a nonlinear programming solver, was used in combination with the DEM solver MUSEN [21]. The fminsearch solver is based on simplex search method proposed by Lagarias et al. [37].

#### 4.2.4. Model Setup

To construct the structural model for the BPM simulation, data obtained from μCT measurements was used. From μCT images, it was observed that the cylindrical samples have a slightly heterogeneous radial porosity distribution. However, this effect was not considered in the model and ideally homogeneous porosity was assumed to generate structural model. The real microstructure of aerogels, consisting of small particles or fibrils with size in the range of 100 Å, cannot be directly reproduced with BPM. The foamed-like structure of material was instead represented as set of primary particles with a radius of 152.5 μm connected with solid bonds.

**Figure 11:**
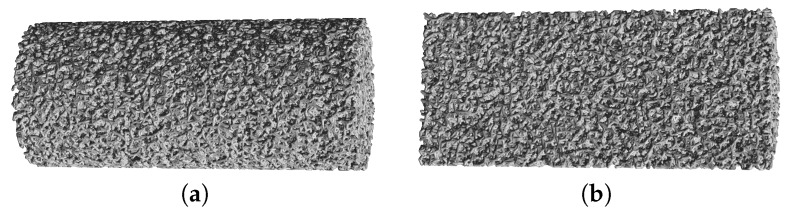
Bonded particle model (BPM) representation of cylindrical aerogel sample (**a**) and its cross-cut (**b**).

In Figure 11 the BPM representation of the initial structure of a cylindrical aerogel sample with a length 27.8 mm and diameter 12.2 mm is shown. This scene consists of 99 thousand primary particles and 380 thousand solid bonds. The average length of solid bond calculated as distance between centers of connected particles is approximately 370 μm.

The parameters of the functional model were adjusted only for the results of the samples that had a calcium chloride concentration of 5 g/L. Application of this procedure resulted in a strain rate of $\epsilon_{y}$ = 3.52% for which the plastic failure starts, a stiffness ratio of β=−0.3 during plastic failure and a stiffness ratio of α=30 during unloading. The resulting stress-strain curve for a concentration of 5 g/L can be seen in Figure 5 in the results chapter. The parameters of interparticle contact, such as sliding or rolling friction coefficient, were not included into the model adjustment strategy and were assumed to be equal to 0.5 and 0.05 accordingly. All parameters are summarised in Table 1.

## Figures and Tables

**Table:** 

	

(**a**)	(**b**)

**Table:** 

		

(**a**)	(**b**)	(**c**)

**Table:** 

		

(**a**)	(**b**)	(**c**)

**Table:** 

	
(**a**)	(**b**)

**Table 1:** BPM parameters used in the study.

Structural Model	
Radius of particles [μm]	152.5
Radius of bonds [μm]	122.5
Particles number	≈99,000
Number of solid bonds	≈380,000
Average bond length [μm]	≈370
**Functional model for bonds**	
Compressive yield strain $\epsilon_{y}$ [%]	3.52
Plastic failure β [-]	−0.3
Interlocking stiffness factor α [-]	30
Poisson ratio	0.2
**Functional model for primary particles**	
Poisson ratio	0.2
Friction coefficient	0.1
Rolling friction	0.05

## Abbreviations

The following abbreviations are used in this manuscript:

BPM	Bonded Particle Model
DEM	Discrete Element Method
μCT	Micro Computed Tomography
